# Tabersonine Induces the Apoptosis of Human Hepatocellular Carcinoma *In vitro* and *In vivo*

**DOI:** 10.2174/0118715206286612240303172230

**Published:** 2024-03-07

**Authors:** Xuan Li, Xudan Li, Lianghua Chen, Yuan Deng, Zhizhong Zheng, Yanlin Ming

**Affiliations:** 1 Department of Bioengineering and Biotechnology, Institute of Chemical Engineering, Huaqiao University, Xiamen, Fujian, 361021, China;; 2 Fujian Provincial Key Laboratory of New Target Drugs (Xiamen University), School of Pharmaceutical Sciences, Xiamen University, Xiamen, 361021, China;; 3 Key Laboratory of Fujian Province for Physiology and Biochemistry of Subtropical Plant, Fujian Institute of Subtropical Botany, Xiamen, 361006, China;; 4 College of Life and Health Sciences, Fuzhou Institute of Technology, Fuzhou, 350506, China

**Keywords:** Tabesonine, anti-tumor, hepatocellular carcinoma, apoptosis, mitochondrial pathway, death receptor apoptotic pathway

## Abstract

**Background:**

Tabersonine, a natural indole alkaloid derived from Apocynaceae plants, exhibits anti-inflammatory and acetylcholinesterase inhibitory activities, among other pharmacological effects. However, its anti-tumor properties and the underlying molecular mechanisms remain underexplored.

**Objective:**

The present study aims to investigate the anti-tumor effects of tabersonine and its mechanisms in inducing apoptosis in hepatocellular carcinoma.

**Methods:**

The inhibitory effects of tabersonine on the viability and proliferation of liver cancer cells were evaluated using MTT assay and colony formation assay. AO/EB, Hoechst, and Annexin V-FITC/ PI staining techniques were employed to observe cell damage and apoptosis. JC-1 staining was used to detect changes in mitochondrial membrane potential. Western blot analysis was conducted to study the anti-tumor mechanism of tabersonine on liver cancer cells. Additionally, a xenograft model using mice hepatoma HepG2 cells was established to assess the anti-tumor potency of tabersonine *in vivo*.

**Results and Discussion:**

Our findings revealed that tabersonine significantly inhibited cell viability and proliferation, inducing apoptosis in liver cancer cells. Treatment with tabersonine inhibited Akt phosphorylation, reduced mitochondrial membrane potential, promoted cytochrome c release from mitochondria to the cytoplasm, and increased the ratio of Bax to Bcl-2. These findings suggested that tabersonine induces apoptosis in liver cancer cells through the mitochondrial pathway. Furthermore, tabersonine treatment activated the death receptor pathway of apoptosis. *In vivo* studies demonstrated that tabersonine significantly inhibited xenograft tumor growth.

**Conclusion:**

Our study is the first to demonstrate that tabersonine induces apoptosis in HepG2 cells through both mitochondrial and death receptor apoptotic pathways, suggesting its potential as a therapeutic agent candidate for hepatic cancer.

## 1 INTRODUCTION

Hepatocellular carcinoma (HCC), a severe malignancy, poses a significant threat to global health. The incidence of HCC exhibits wide variations across geographical regions, with Asia and Africa reporting higher rates [1]. Recently, there has been an increase in the incidence of liver cancer, particularly advanced HCC, ranking fifth globally and third in terms of fatality rate [2, 3].

HCC is a complex malignancy characterized by diverse signal pathway alterations, challenging its treatment [4]. Current chemotherapeutic options offer limited efficacy and often come with significant toxicity-related side effects [5]. Many patients are diagnosed at advanced, incurable stages, rendering surgical intervention unfeasible.

Fortunately, the situation is gradually improving. Comprehensive research into the molecular mechanisms underlying HCC progression has led to a deeper understanding of the disease. This, in turn, has sparked interest in natural products as potential agents for targeted HCC therapy [6]. Ongoing studies are exploring the therapeutic potential of natural compounds, aiming to develop more effective and less toxic treatment options for HCC patients. While much work remains to be done, these advances offer hope for improved outcomes and survival rates for those affected by this devastating disease.

**Fig. (1):**
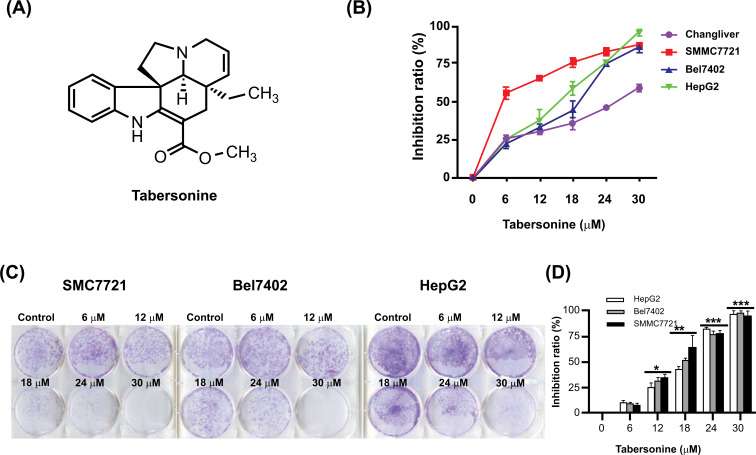
Inhibition of viability by tabersonine in hepatocellular carcinoma cells. (**A**) The chemical structure of tabersonine; (**B**) The cytotoxic effect of tabersonine on hepatocellular carcinoma cell lines SMMC7721, Bel7402, and HepG2 and normal liver cell line Changliver; (**C**, **D**) The inhibitory effect of tabersonine on colony formation in SMMC7721, Bel7402, and HepG2 cell lines. n = 3 per group. *, *p* < 0.05, **, *p* < 0.01, ***, *p* < 0.001, compared with the control group.

Tabersonine (C_21_H_24_N_2_O_2_) (Fig. **1A**) is a natural indole alkaloid mainly isolated from the medicinal plant *Voacango africana* [7], *Amsonia tabernaemontana* [8], *Cantharanthus roseus* [9]. Despite the scarcity of research on the bioactivity of tabersonine, studies have begun to reveal its diverse therapeutic benefits. To date, tabersonine has been shown to possess anti-inflammatory [10, 11], acetylcholinesterase inhibitory activity [12], improve atherosclerosis [13], prevention of osteoporosis [14] and aid in the improvement of spinal cord injuries [15].

Notably, tabersonine is a key precursor in synthesizing vinblastine, a potent anticancer compound [16]. Although its direct anticancer effects are not fully understood, the previous study by our team has shown that tabersonine exhibits broad-spectrum cytotoxicity against tumor cells and maybe through the induction of apoptosis [17]. Importantly, tabersonine has shown a good anti-tumor effect on liver cancer, highlighting its potential as an anti-tumor agent. In addition, considering that tabersonine is a precursor of vincristine used in clinical tumor treatment, the smaller molecules make it easier to utilize and a potential anti-tumor drug candidate. Therefore, in this study, we aim to comprehensively investigate the effects of tabersonine on liver cancer cells and animals, seeking to understand its potential therapeutic benefits and mechanisms and contributing to developing new and effective anticancer strategies.

## 2 METHODS

### 2.1 Antibodies and Reagents

Tabersonine (≥98% purity) was previously prepared in our laboratory. HepG2, SMMC7721, Bel7402 and Changliver were procured from the Cell Bank of the Chinese Academy of Sciences (Shanghai, China). The RPMI-1640 and DMEM medium were obtained from Hyclone (Grand Island, NY, USA). MTT (≥98% purity) and cisplatin (CDDP; ≥99.9% purity) were obtained from Sigma (St. Louis, MO, USA). Bisben zimide (Hoechst 33258), acridine orange (AO), ethidium bromide (EB), JC-1 reagents, Annexin V-FITC-PI Apoptosis Detection Kit, LY294002 (≥98% purity), Cocktail100x of proteinase and phosphatase inhibitors were purchased from Roche Ltd. Polyclonal antibodies specific to anti-Caspase-3 (9662S), anti-Cleaved Caspase-3 (9664T), anti-PARP (9532T), anti-Bcl-2 (15071S), anti-Bax (5023), Bid (8762S) anti-cytoc (11940S), anti-Caspase-9 (9504T), anti-Caspase-8 (9746T), anti-Fas (8023S), anti-FasL (68405S), anti-Akt (4691S), anti-p-Akt (Ser473) (4060S), Tom20 (42406S), Tubulin (2148S) were purchased from Cell Signaling Technology (Danvers, MA, USA).

### 2.2 Cell Culture and Tabersonine Treatment

HepG2, SMMC7721, Bel7402 and Changliver cells were cultured in RPMI-1640 or DMEM medium with 10% FBS in a humidified incubator (5% CO_2_, 37℃) as described previously [16]. 50 mM tabersonine was dissolved in DMSO for storage and diluted with RPMI-1640 or DMEM medium to final concentrations of 6, 12, 18, 24, and 30 μM as the experimental group in each experiment and drug-free culture medium as a control group. Normal liver cell line Changliver is used as a control group in the MTT assay.

### 2.3 MTT Assay

1×10^5^ cells/ well were seeded in 96-well plates and cultured in a humidified incubator (5% CO_2_, 37℃) for 24 h. Then, tabersonine was added to each well with different concentrations and cells were cultured in a humidified incubator (5% CO_2_, 37℃) for 24 h. DMSO was added to the control group, and three replicates were set for each group. After treatment, 100 μL 5 mg/mL MTT was added to each well and incubated in a humidified incubator (5% CO_2_, 37℃) for 4 h. Finally, the Medium was removed, and DMSO was added to dissolve the products fully. A microplate reader detected the cell's viability at 450 nm.

### 2.4 Colony Formation Assay

1.5×10^6^ cells/ well were seeded in 6-well plates and cultured in a humidified incubator (5% CO_2_, 37℃) for 24 h. Then, cells were treated with 3 mL of the solution of tabersonine to be tested (0, 6, 12, 18, 24, 30 μM) for 24 h. The experimental procedure involved replacing the culture medium with a fresh dose every 3 days, discontinuing the culture after two weeks of continuous growth, and discarding the old culture solution. Subsequently, each well was fixed with an appropriate concentration of 4% paraformaldehyde and 0.2% crystal violet for 30 minutes. Following fixation, the bottom of each well in the 6-well plate was scanned using a scanner. The number of colonies having a diameter greater than 75 μm was counted, and the formula of colony inhibition rate (%) = (1 - colony number administration ÷ colony number control) × 100%.

### 2.5 Morphological Study

According to the MTT assay, cells were seeded and treated with 0, 6, 12, 18, 24, 30 μM tabersonine and 30 μM CDDP as a positive control for 24 h. 100 μg/mL staining was added in each well of the plates for 10 minutes in light-deprived conditions. The cell morphology was observed by fluorescence microscopy (AMG EVOS, USA).

### 2.6 Mitochondrial Membrane Potential Assay

According to the MTT assay, cells were seeded and treated with 0, 6, 12, 18, 24, 30 μM tabersonine and 30 μM CDDP as a positive control for 24 h. 100 μl 5 μg/mL JC-1 was added to each well for 30 minutes, and cell morphology was then visualized using fluorescence microscopy (AMG EVOS, USA).

### 2.7 Annexin V-FITC/ PI Apoptosis Detection

After the cells reached complete adherence, the cells were treated with 0, 6, 12, 18, 24, 30 μM tabersonine solution for 18 h. After incubating the cells with 10 μl Annexin V-FITC for 10 minutes, 5 μL of PI was added, mixed in light-deprived conditions at 25℃ for 15 minutes and detected by flow cytometry (Thermo, Attune NxT) within 1 h. Before the machine testing, the mix was filtered with a 200-300 mesh filter.

### 2.8 Western Blot

After treatment with tabersonine (6, 12, 18, 24, 30 μM), the cells were harvested and washed twice with PBS. The cells treated with RIPA lysis buffer contained proteinase inhibitor, phosphatase inhibitor and phenylmethanesulfonyl fluoride (PMSF) on ice for 30 minutes. Then, cells were centrifuged at 12000 g for 10 minutes (4℃). The supernatants were collected and used for protein content detection and SDS-PAGE. Proteins were transferred onto the PVDF membrane, and the blots were incubated with different antibodies. Enhanced chemiluminescence was added to the membrane for exposure imaging (Bio-Rad).

### 2.9 Animal Experiments

All animals were purchased from the Xiamen University Laboratory Animal Center, and all animal experiments were approved by the Experimental Animal Ethics Committee of Xiamen University (No. XMULAC20210054). 2×10^7^ HepG2 cells were injected in male BALB/c nude mice. After three days, tabersonine (25 mg/kg, 50 mg/kg) was administered by gavage for three consecutive weeks, and the volume of tumor tissue was measured every three days. After three weeks of treatment with tabersonine, the mice were euthanized, and tumors were collected for tumor volume and immunofluorescence staining analysis. The frozen section was immunofluorescent staining with antibodies against cleaved Caspase-3 and observed by Laser Scanning Confocal Microscope (Zeiss).

### 2.10 Statistical Assay

The GraphPad Prism 6.0 and SPSS 17.0 software were used for data processing and statistical analysis.

The significant analysis was performed using one-way ANOVA or two-sided unpaired Student’s t-test. *p* < 0.05 was considered statistically significant. The results were presented as mean ± standard deviation.

## 3 RESULTS

### 3.1 Tabersonine Inhibits the Growth of HCC Cells

To assess the cytotoxic effects of tabersonine on hepatocellular carcinoma cells, an MTT assay was conducted across various concentrations (6, 12, 18, 24, 30 μM) in three distinct HCC cell lines: SMMC7721, Bel7402, and HepG2. The results showed that tabersonine could inhibit the viability of SMMC7721, Bel7402, and HepG2 cell lines, with the IC_50_ values of 7.89 ± 1.2, 5.07 ± 1.4, 12.39 ± 0.7 μM, respectively (Fig. **1B**). These findings suggested that tabersonine had a potent inhibitory effect on HCC cell viability. A clone formation assay was employed to investigate the antiproliferative activity of tabersonine further. As shown in Figs. (**1C** and **D**), tabersonine significantly inhibited the formation of clones in all three HCC cell lines with the concentration range from 12 to 30 μM. This inhibition of clone formation further confirmed the antiproliferative effects of tabersonine on HCC cells. The results above indicated that tabersonine had a significant effect of inhibition on the viability of HCC cells.

### 3.2 Tabersonine Induces the Apoptosis of HepG2 Cell

**Fig. (2):**
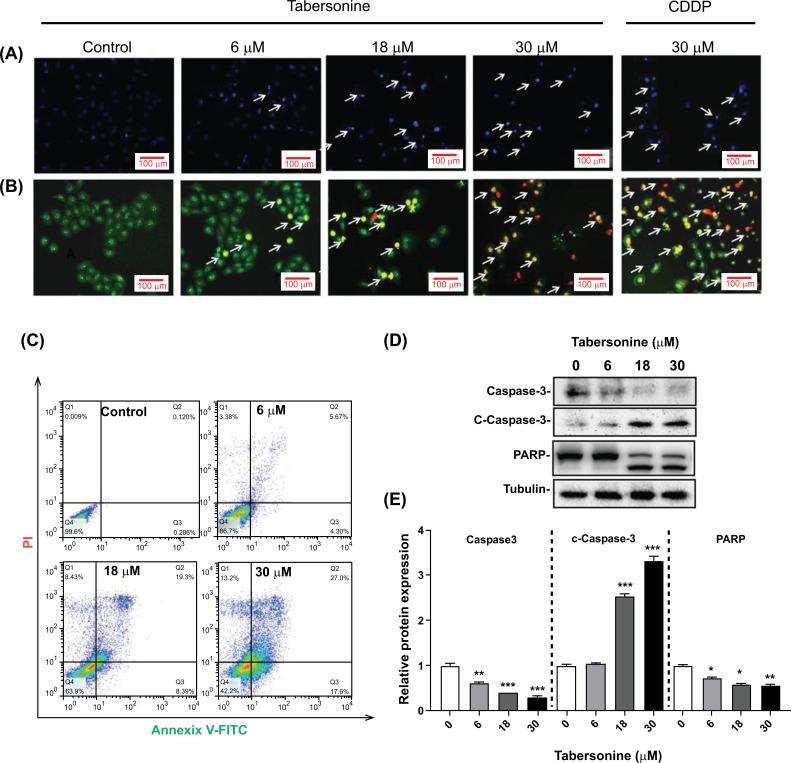
Apoptosis-inducing effect of tabersonine on human hepatoma cell HepG2. (**A**) Hoechst 33258 staining of HepG2 treated with tabersonine, arrows marked the apoptotic cells with bright staining of nuclear shrinkage (200 x); (**B**) AO/EB staining of HepG2 treated with tabersonine, arrows marked the apoptotic cells with different staining changes (200 x); (**C**) Annixian V/PI staining of HepG2 treated with tabersonine; (**D**) Western blot for Caspase-3, c-Caspase-3 and PARP; (**E**) Semi-quantitative analysis for Caspase-3, c-Caspase-3 and PARP. n = 3 per group. *, *p* < 0.05, **, *p* < 0.01, ***, *p* < 0.001, compared with the control group.

To further elucidate the cytotoxic mechanisms of tabersonine against liver cancer, the present study focused on HepG2 cells, a widely used model for hepatocellular carcinoma. Morphological analysis using Hoechst 33258 and AO/EB staining revealed characteristic apoptotic features in HepG2 cells treated with tabersonine. The cells showed nucleus shrinkage or fragmentation (Figs. **2A** and **B**). Flow cytometry was employed using Annixian V/PI staining for more quantitative analysis. In the experiment, the signals of Annixian V/PI staining (Fig. **2C**) evaluated the degree of apoptosis, and the apoptotic rate reached 27% with 30 μM tabersonine treatment. Moreover, other apoptotic hallmarks, cleavage of Caspase-3 and poly ADP-ribose polymerase (PARP), were observed (Figs. **2D** and **E**). These results above indicated that tabersonine could induce apoptosis in HepG2 cells.

### 3.3 Tabersonine Induces Mitochondrial Dysfunction in HepG2 Cell

**Fig. (3):**
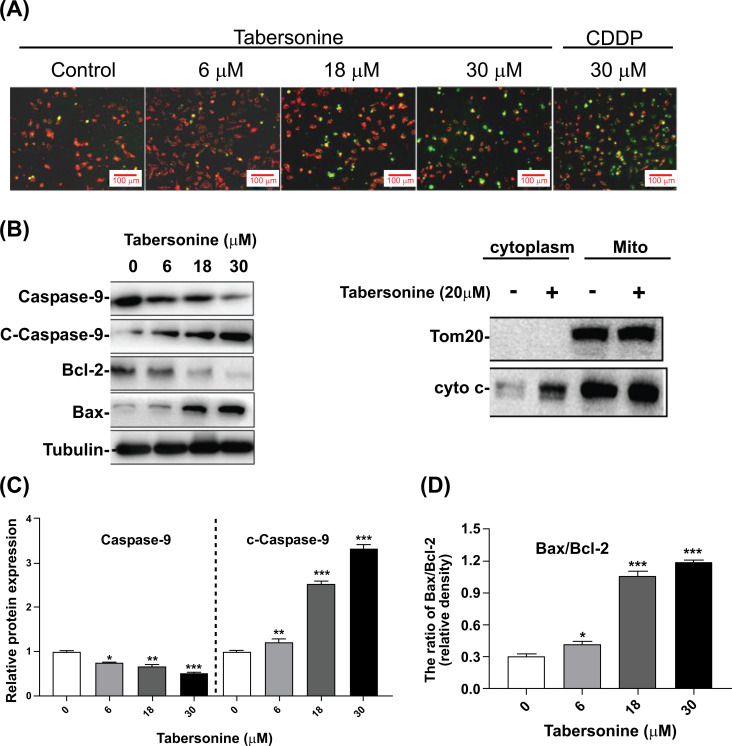
Effects of tabersonine on mitochondrial function in HepG2 cells. (**A**) JC-1 staining of HepG2 treated with tabersonine (200 x); (**B**) Western blot and semi-quantitative analysis for mitochondrial apoptotic pathway-related proteins; (**C**) Semi-quantitative analysis for Caspase-9 and c-Caspase-9; (**D**) Semi-quantitative analysis for the ratio of Bax/Bcl-2. n = 3 per group. *, *p* < 0.05, **, *p* < 0.01, ***, *p* < 0.001, compared with the control group.

Mitochondrial-mediated apoptotic pathway is a classic apoptotic pathway in programmed cell death. This study found that tabersonine could reduce the mitochondrial membrane potential (Δψm) in HepG2. As shown in Fig. (**3A**), JC-1 was accumulated in HepG2 cells (red) and poorly accumulated in tabersonine-treated cells (green), which indicated Δψm in HepG2 cells change from high potential to low potential with a dose-dependent tabersonine treatment. Moreover, the ratio of Bax/Bcl-2 was significantly increased, and the cytochrome c in the cytoplasm increased, activating the cleaved-Caspase-9 protein (Figs. **3B**-**D**). The above results indicate that tabersonine can trigger apoptosis in HepG2 cells by disrupting mitochondrial membrane potential, modulating the Bax/Bcl-2 ratio, promoting cytochrome c release, and activating the mitochondria-mediated endogenous apoptotic pathway.

### 3.4 Tabersonine Inhibited the PI3K/Akt Pathway

**Fig. (4):**
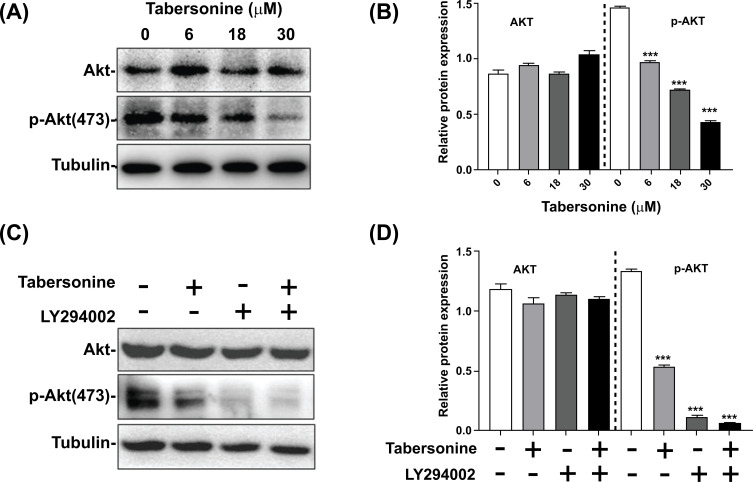
Effect of tabersonine on the PI3K/Akt signaling pathway in HepG2 cells. (**A**) Western blot for Akt and p-Akt without PI3K inhibitor; (**B**) Semi-quantitative analysis for Akt and p-Akt without PI3K inhibitor; (**C**) Western blot for Akt and p-Akt with PI3K inhibitor LY294002; (**D**) Semi-quantitative analysis for Akt and p-Akt with PI3K inhibitor LY294002. n = 3 per group. ***, *p* < 0.001, compared with the control group.

The PI3K/Akt signaling pathway is a critical regulator of the apoptosis signaling pathway, and activation of the PI3K/Akt pathway can promote the proliferation and survival of cancer cells. In this study, we investigated the effects of tabersonine on Akt phosphorylation and its interaction with the PI3K inhibitor LY294002. The results showed that tabersonine downregulated the expression of p-Akt and blocked the phosphorylation of Akt, but tabersonine did not affect the expression of total Akt (Figs. **4A** and **B**). This suggested that tabersonine may specifically target Akt phosphorylation without altering the overall Akt protein level. Subsequently, LY294002 (PI3K inhibitor) was used to observe the targeted role of tabersonine. As shown in Figs. (**4C** and **D**), LY294002 and tabersonine markedly inhibited the expression of p-Akt, while the expression of Akt was unchanged. Compared with treatment with LY294002 or tabersonine alone, treatment with LY294002 and tabersonine showed a more pronounced inhibition of p-Akt expression, which indicated that tabersonine and LY294002 act synergistically to block Akt phosphorylation. Our findings indicated that tabersonine is a potent inhibitor of Akt phosphorylation and can enhance the inhibitory effects of LY294002.

### 3.5 Tabersonine Activates the Death Receptor-mediated Apoptosis Pathway

**Fig. (5):**
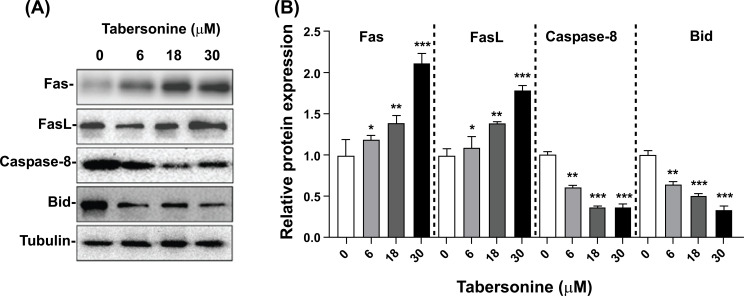
Tabersonine involved expression of the protein on death receptor-mediated extrinsic pathway. (**A**) Western blot for Fas, Fas-L, Caspase-8 and Bid; (**B**) Semi-quantitative analysis for Fas, FasL, Caspase-8 and Bid. n = 3 per group. *, *p* < 0.05, **, *p* < 0.01, ***, *p* < 0.001, compared with the control group.

The death receptor pathway is a major apoptotic pathway regulating cell death. In this study, we investigated the effect of tabersonine on the death receptor pathway in HepG2 liver cancer cells. As depicted in Fig. (**5A**), treatment with tabersonine significantly increased the Fas and FasL expression levels. Concurrently, the expression levels of Caspase-8 and Bid were significantly decreased. In the death receptor pathway, FasL binds to its corresponding receptor, FAS, to activate the receptor and induce the binding of Fas intracellular domain with Fas-related protein FADD. FADD activated downstream Caspase-8, which led to a decrease of Caspase-8 protein level and an increase of cleaved-Caspase-8 and activated the death receptor-mediated apoptosis pathway. Therefore, our findings suggested that tabersonine could activate the death receptor pathway to induce apoptosis in HepG2 cells (Fig. **5**).

### 3.6 Effect of Tabersonine on Tumor Growth and Apoptosis in Animals

**Fig. (6):**
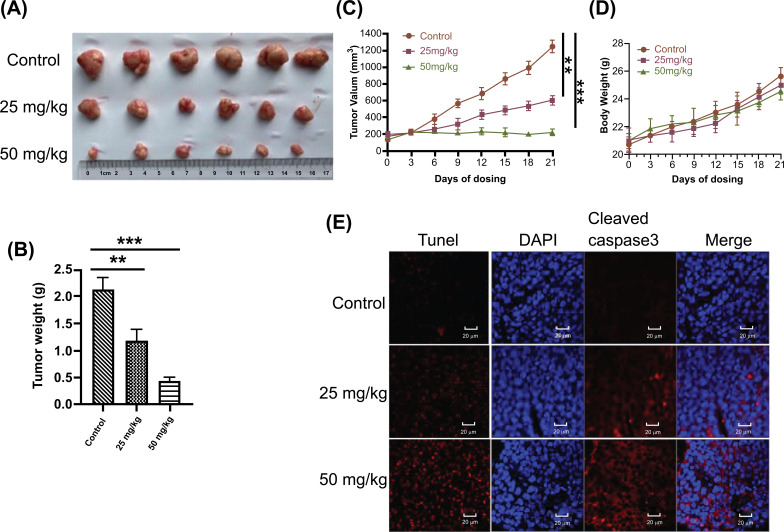
Effect of tabersonine on tumor growth and apoptosis in animals. (**A**, **B**) Tabersonine exhibited anti-tumor efficacy in a xenograft mouse model of HepG2 cells; (**C**, **D**) Body weight and tumor volume of nude mice during the experimental period; (**E**) Tunel staining and immunofluorescence staining in tumor tissue. n = 6 per group. **, *p* < 0.01, ***, *p* < 0.001, compared with the control group.

Further, the animal experiment was conducted to evaluate the anti-tumor effect of tabersonine *in vivo*. As shown in Fig. (**6A**), tabersonine treatment significantly inhibited tumor growth compared to the control group. This was further confirmed by the reduction in tumor weight observed in Fig. (**6B**). Treatment with 25 mg/kg/day and 50 mg/kg/day of tabersonine effectively suppressed tumor growth without causing any significant effect on the overall growth of the mice as evident from Figs. (**6C** and **D**). To understand the mechanism underlying the anti-tumor effects of tabersonine, we performed Tunel staining, a marker for apoptotic cells. Compared with the control group, the apoptosis of tumor tissue cells was increased (Fig. **6E**). This indicated that tabersonine induced apoptosis in liver cancer cells. In addition, immunostaining of tumor tissues showed the expressions of cleaved Caspase-3 were upregulated in the tabersonine-treated group (Fig. **6E**). The upregulation of cleaved Caspase-3 further confirmed the apoptotic effect of tabersonine *in vivo*. Taken together, these results strongly suggested that tabersonine had significant anti-tumor effects *in vivo* and could effectively inhibit the growth of liver cancer in a nude mouse model.

## 4 DISCUSSION

Liver cancer remains a significant health concern worldwide due to its high mortality and recurrence rates [18]. Although there has been significant progress in the treatment of liver cancer both domestically and abroad, conventional treatment approaches are becoming increasingly constrained in the course of treatment due to their conditional restrictions and harmful side effects [19]. This underscores the urgent need for exploring novel therapeutic approaches that can effectively target liver cancer while minimizing side effects. In recent years, there has been growing interest in using natural products, particularly those derived from plants or the ocean, as potential sources of new cancer treatments. These natural compounds have shown promising medical effects with minimal negative side effects, making them attractive candidates for further research and development [20].

Previous research has established that alkaloids, a class of compounds, can induce liver cancer cell death by arresting the cell cycle and triggering apoptosis and necrosis [21]. The current study employed various techniques to investigate the anticancer effects and mechanisms of tabersonine. These included AO/EB, Heochst33258 staining, Annexin V-FITC/PI, western blot analysis and animal experiments. These methods revealed that tabersonine induced apoptosis in HepG2 cells dose-dependently. The findings of this study are significant as they provide further evidence for the potential use of natural products in cancer treatment.

Apoptosis, or programmed cell death, is critical to maintaining cellular homeostasis. Major apoptosis pathways include death receptor-mediated exogenous apoptosis and endogenous apoptosis routes mediated by mitochondria [22]. These pathways converge on the activation of caspases. Our study found that tabersonine can induce apoptosis of HepG2 cells through the mitochondrial and death receptor apoptosis pathways. This dual mechanism of action suggested that tabersonine had a robust ability to trigger cell death in liver cancer.

On the one hand, tabersonine was found to regulate the Bax/Bcl-2 ratio, increase mitochondrial membrane permeability, and promote cytochrome c release. These events are key steps in the mitochondrial apoptotic pathway. Additionally, tabersonine promoted the release of cytochrome c and activation of caspase-9, which indicated that it triggered apoptosis through the mitochondrial pathway. This mechanism of action is consistent with its anticancer properties, as apoptosis is a critical process in eliminating cancer cells.

On the other hand, the Fas/FasL system is the main signal transduction pathway in cell and tissue apoptosis. Anticancer drugs activate soluble FasL and bind to Fas in autocrine and/or paracrine forms [23]. In addition, membrane FasL and Fas are simultaneously activated, leading to FasL binding to Fas between the same cell and adjacent cells [24]. FasL binding to Fas leads to activation of initiation Caspase-8 and Caspase-10, which directly activate precursor proteins caspase-3 and Caspase-7 [25]. Caspase-3 is a critical executive protein in the process of cell apoptosis. Excited Caspase-3 induces cell apoptosis by acting on the substrate PARP [26]. Our study showed that Fas, FasL and cleaved Caspase-8 expressions were significantly up-regulated after tabersonine treatment for 24 h. These results suggested that the induction of apoptosis by tabersonine in HepG2 cells may be related to the death receptor pathway.

Another key finding of the study was that the protein level of Bid was decreased, which may indicate the cleavage of Bid, as cleaved-Bid could induce the release of cyto c from mitochondria to the cytoplasm. This cytochrome c release leads to the activation of Caspase-3, a key executioner of caspase in the apoptotic cascade. Therefore, Bid, as a bridging molecule of mitochondrial and death receptor apoptotic pathway, can transduce Fas-mediated apoptosis signals into the mitochondrial apoptotic pathway and enhance the apoptosis-inducing ability of tabersonine. It is speculated from the experimental results that the cleavage of Bid protein induced by tabersonine can transmit a Fas-mediated apoptosis signal to the mitochondrial pathway.

In addition, The PI3K/Akt signaling pathway plays a pivotal role in regulating cellular processes such as proliferation, survival, and apoptosis [27]. Blocking the PI3K/Akt signaling pathway has become an effective method for intervention in cancer [28]. Tabersonine can suppress Akt phosphorylation in a dose-dependent manner. This inhibition of Akt phosphorylation suggested that tabersonine may interfere with the activation of the PI3K/Akt pathway, leading to apoptosis in HepG2 cells. To validate these findings further, we combined tabersonine treatment with LY294002. The results showed that the combination treatment further inhibited Akt phosphorylation, indicating a synergistic effect between the two compounds in blocking the PI3K/Akt pathway. This synergistic inhibition of Akt phosphorylation suggested that tabersonine may potentiate the anticancer effects of LY294002 or other PI3K inhibitors.

Importantly, the *in vivo* experiments conducted in this study demonstrated that tabersonine significantly inhibited the growth of xenograft tumors derived from liver cancer cells. Tunel staining and immunofluorescence staining both supported the apoptotic mechanism induced by tabersonine. These data further supported the anticancer potential of tabersonine.

## CONCLUSION

The study investigated the potential of tabersonine, a naturally occurring alkaloid, as a novel anti-tumor drug for liver cancer. According to this study, tabersonine can induce apoptosis of liver cancer cells through the death receptor and mitochondrial pathways, indicating its ability to trigger multiple cellular mechanisms leading to tumor cell death. The apoptotic effect of tabersonine on liver cancer cells suggests it has potential as an anti-tumor drug. However, it is essential to note that while these results are promising, further research is needed to evaluate the potential of tabersonine in liver cancer treatment and to clarify its mechanism of action. In conclusion, tabersonine holds significant promise as a potential new medication for treating liver cancer.

## Data Availability

The corresponding author can obtain the present study's data and related materials.

## LIST OF ABBREVIATIONS

HCC: Hepatocellular Carcinoma
PARP: Poly ADP-ribose Polymerase
